# Anti-inflammatory Role of Trypsin, Rutoside, and Bromelain Combination in Temporomandibular Joint Osteoarthritis: A Systematic Review

**DOI:** 10.7759/cureus.51749

**Published:** 2024-01-06

**Authors:** Amlendu Shekhar, Nisha Maddheshiya, Adit, Varun Rastogi, Karthikeyan Ramalingam

**Affiliations:** 1 Oral Medicine and Radiology, Faculty of Dental Sciences, Institute of Medical Sciences, Banaras Hindu University, Varanasi, IND; 2 Oral Pathology, Universal College of Medical Sciences and Teaching Hospital, Bhairahawa, NPL; 3 Oral Pathology and Microbiology, Saveetha Dental College and Hospitals, Saveetha Institute of Medical and Technical Sciences, Saveetha University, Chennai, IND

**Keywords:** systemic enzyme therapy, pain, internal derangement, osteoarthritis, tmj diseases, systematic review, nsaids, rutoside, bromelain, trypsin

## Abstract

The objective of this systematic review was to assess the effectiveness, acceptability, and safety of systemic enzyme therapy, consisting of trypsin, bromelain, and rutoside trihydrate, as an anti-inflammatory agent, either when utilized independently or in conjunction with non-steroidal anti-inflammatory drugs (NSAIDs). This systematic review adhered to the Preferred Reporting Items for Systematic Reviews and Meta-Analyses (PRISMA) guidelines. Two studies met the inclusion criteria and were assessed in the review. The bias risk was evaluated using the risk-of-bias tool for randomized trials (RoB 2). Both studies revealed highly significant results for the study population. Individuals receiving oral enzymes and diclofenac sodium combination therapy showed a significant improvement in pain reduction, better eating, and mouth opening, as well as a decrease in joint noise and jerky mandibular motions. Patients receiving systemic enzyme therapy with diclofenac combinations performed better than those receiving NSAIDs alone, and the differences were quite substantial. For the treatment of internal derangement of the temporomandibular joint (TMJ), we recommend combining enzymes and diclofenac. Systemic enzyme therapy can be used in the treatment of TMJ osteoarthritis, as it shows a highly significant result in the study population.

## Introduction and background

Oral use of non-steroidal anti-inflammatory drugs (NSAIDs) is clinically proven to be quite effective for treating osteoarthritis pain. Non-selective or cyclooxygenase (COX)-2-selective NSAIDs are first-line therapies for pain relief. Their choice is determined by their safety profiles, patient-specific risk factors, and coupled ailments to increase their tolerability. While generally safe at low doses, NSAIDs, whether COX2-selective or not, are typically utilized in the short term. They are often associated with adverse side effects, and the risk of significant complications involving the cardiovascular, renal, and gastrointestinal (GI) systems increases, especially when administered over an extended period, at higher doses, and in conjunction with other illnesses or in elderly patients. This scenario is frequently encountered in temporomandibular joint (TMJ) osteoarthritis patients [1, 2, 3].

The application of proteolytic enzymes orally, known as systemic enzyme therapy, combines digestive enzymes like trypsin and bromelain with antioxidants like rutoside. In the small intestine, enzymes are absorbed and at least partially absorbed into the bloodstream [4]. It also has an anti-inflammatory effect similar to serine protease trypsin [5]. Recent studies imply that there is a need for more alternatives that are natural and secure for managing pain and swelling. Several studies have been conducted on essential fatty acids, ginger, proteases (trypsin, bromelain), bioflavonoids, turmeric, and *Boswellia *(rutoside). Clinical trials demonstrate the effectiveness and safety of the bromelain, trypsin, and rutoside fixed-dose combination in treating edema and inflammation and facilitating the healing of wounds [2, 5]. Similar to this, the pineapple stem cysteine protease bromelain is a potent phytotherapeutic medication with anti-inflammatory characteristics [6, 7]. Rutin, a flavonoid also known as rutoside, is another potential component of systemic enzyme therapy and is recognized for its cytoprotective and anti-inflammatory properties [8-13].

Temporomandibular joint osteoarthritis is a degenerative condition with an unknown etiology. Its symptoms include pain around the jaws, especially during movements. It is identified by physical and radiological examination. There is no definite cure except for symptomatic management. We know that pain is a complex symptom caused by a multitude of etiological factors. Direct proteolytic and thrombolytic action may also play a role in how proteolytic enzymes modulate immune responses in several immunomodulatory pathways [7]. These systems consist of interaction with trypsin-activated receptor 2 (PAR-2), a protease, on some immune system components such as macrophages that attach to alpha-2 macroglobulin, after which the linked cytokines are cleared. Proteases have also been proposed to contribute to enhanced blood fluidity.

The efficiency of enzyme therapy depends on the specific type of pain and its underlying cause. Limited literature is available on the management of TMJ osteoarthritis and no systematic review has been conducted to date. Hence, this study aimed to assess the effectiveness, acceptance, and safety of systemic enzyme therapy, including trypsin, bromelain, and rutoside trihydrate, as anti-inflammatory agents, whether independently or in combination with conventional NSAIDs for the management of TMJ osteoarthritis.

## Review

Data source selection 

This systematic review adhered to the Preferred Reporting Items for Systematic Reviews and Meta-Analyses (PRISMA) guidelines. 

Search strategy 

The level of agreement between the reviewers was determined through Cohen's kappa statistical analysis, yielding a value of 0.91. The data that were extracted included full text. Two reviewers conducted electronic systematic literature searches independently. For the current analysis, the metadata utilized for article searches included databases such as PubMed, Medical Literature Analysis and Retrieval System Online (MEDLINE), Cochrane, Web of Science, Scopus, and Google Scholar. In PubMed, medical subject headings (MeSH) were employed to identify terms akin to enzymes. Filters were implemented during the search, focusing on criteria such as study time frame, study design, geographical location, and language. Various combinations of keywords were employed across the mentioned search engines. Data were collected from the electronic databases using the keywords “systemic enzyme therapy” OR "trypsin,” "bromelain,” “rutoside trihydrate” AND “efficacy” “tolerability” with the Boolean operators “AND” and “OR”. Literature published from January 2017 to August 2022 was included in the screening process. Snowballing and reverse snowballing were done to eliminate any chance of missed articles. The complete text of the approved article was extracted and evaluated to confirm the relevance of its contents for inclusion in the systematic review. The study characteristics (country, year of publication, and study type), participant characteristics, and outcomes were reported.

Eligibility criteria 

The eligibility of selected articles was assessed by screening their titles and abstracts. Full texts were retrieved and evaluated for those studies fulfilling the inclusion criteria. Relevant data were extracted. One author performed all the literature selection steps and then discussed the differences with a second author. The efficacy, tolerability, and safety of trypsin were evaluated and compared with bromelain and rutoside trihydrate therapy.

Articles fulfilling the following criteria qualified for the in-depth analysis: randomized controlled trials including the three drugs; articles published between 2017 and 2022; and articles published in the English language only. Exclusion criteria were articles not containing a clear-cut description of systemic enzyme findings, animal studies, expert opinions, narrative reviews, and literature reviews.

Quality evaluation 

**Figure 1:**
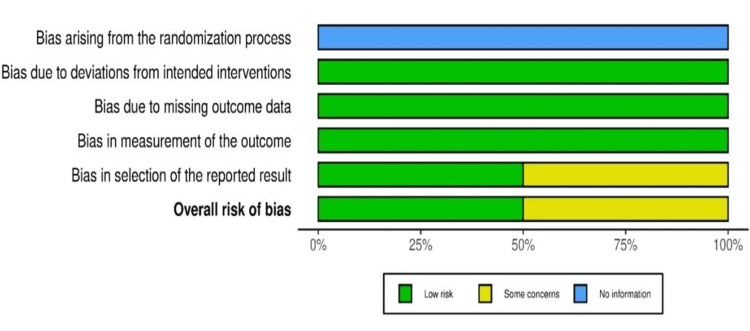
Risk of bias assessment according to the study characteristics

The risk of bias assessment was carried out according to the study characteristics (Figure 1).

**Figure 2:**
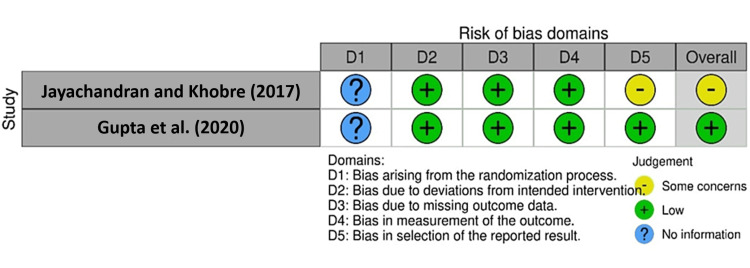
Risk of dias domains Risk of bias domains evaluated for [14, 15]

Two reviewers for randomized controlled trials evaluated these studies using the risk-of-bias tool for randomized trials (RoB 2) (Figure 2).

The tool incorporates signaling questions aimed at extracting information regarding critical aspects of the studies, particularly concerning the bias risk. Studies were categorized as having a "low," "moderate,” “some concerns,” “no information,” or “high” bias risk. Any discrepancy in the quality evaluation was resolved via discussion.

The PRISMA flow chart 

Between March 2022 and May 2022, a total of 3,113 articles from PubMed, Medline, Scopus, Web of Science, Google Scholar, and Cochrane were extracted. Title-based articles were considered for the initial review. Endnote software (Clarivate, London, UK) was used to remove duplicate articles, resulting in 981 unique articles considered for further examination in the study. After the initial screening, 301 articles identified as review articles were excluded. Additionally, 37 articles written in a language other than English were removed. Subsequently, 373 articles were subjected to a detailed analysis of their titles. Those articles deemed relevant to the topic were retained for further analysis in subsequent stages of the study.

**Figure 3:**
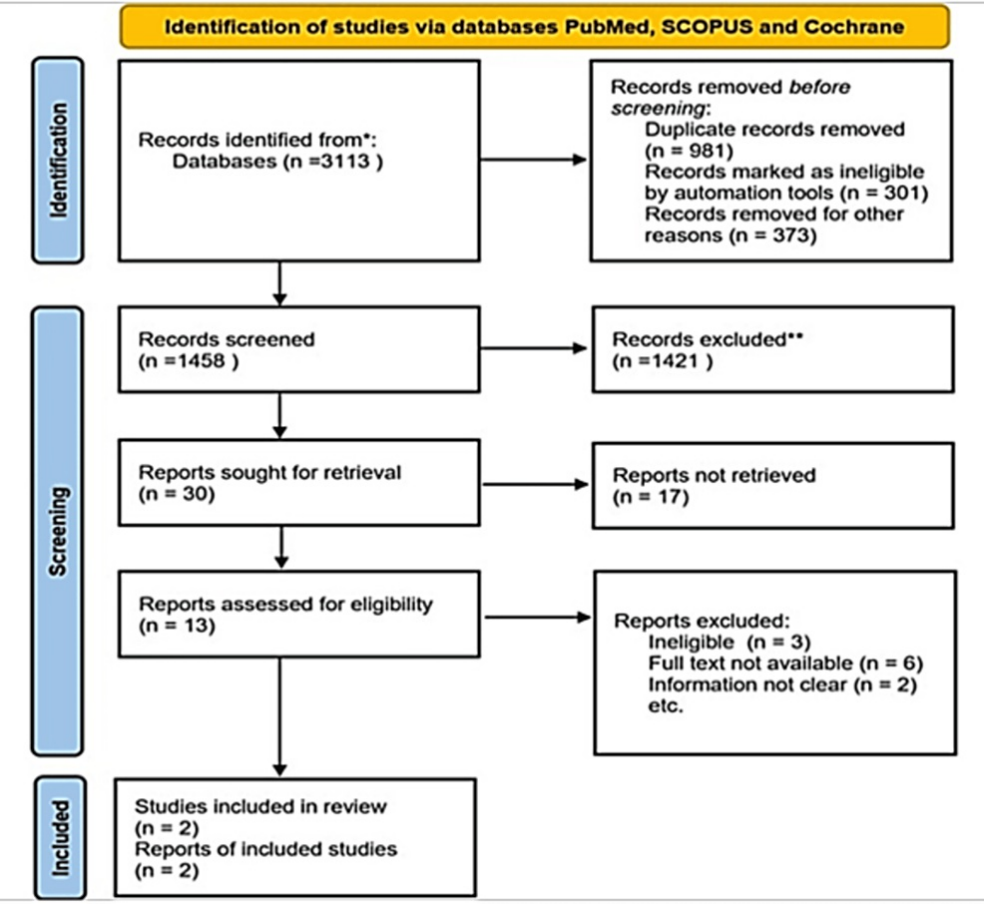
A PRISMA flowchart outlining the identification, screening, and selection of articles for this systematic review PRISMA: Preferred Reporting Items for Systematic Reviews and Meta-Analyses

The articles were scrutinized based on the population, intervention, control, and outcomes (PICO) format, resulting in the exclusion of 190 articles due to a lack of conceptual relevance. Additionally, 41 articles were removed as they did not meet the specified inclusion criteria. Two articles that aligned with the PICO format and met the eligibility criteria for the review were selected for qualitative analysis. In instances where doubts arose regarding the inclusion process, expert opinion was consulted to resolve any discrepancies. The details of the search strategy and inclusion process are further illustrated in the PRISMA flow chart (Figure 3).

Results 

The present systematic review included two randomized clinical trials with patients suffering from TMJ osteoarthritis. Both studies showed significant results with systemic enzymes. The studies used a numerical pain scale to evaluate the efficiency of the drug (Table 1).

**Table 1:** Study characteristics of the two assessed studies

Study ID	Sample size	Trial arm	Randomization	Blinding	Scale	Result
Jayachandran S, Khobre P [14]	30 patients divided into three groups of 10 patients in each group	Multiple arms	Not mentioned	Not done	The pain was recorded on the numeric pain scale	Statistically significant
Gupta P et al [15]	30 patients divided into two groups of 15 patients in each group	Double arm trial	Not mentioned	Not mentioned	The pain was recorded	Statistically significant

Population 

Jayachandran and Khobre chose patients with TMJ osteoarthritis who had symptoms such as pain with mandibular movement or at rest, crepitation, and restriction of mouth opening. Subsequently, they sought evidence of TMJ osseous changes in radiographs to validate the diagnosis. The study excludes patients with myelogenous causes of pain, ankylosis, recent histories of TMJ trauma or surgery, peptic ulcer history, drug allergies, and pregnant women [14]. In 2020, Gupta et al. conducted a study on patients experiencing TMJ pain with decreased mouth opening (normal range: 35 mm to 50 mm), restricted laterotrusive movements (normal range: 5 mm to 10 mm for tips and contralateral joint movements), TMJ noise, intermittent joint lock, and deflection or deviation of the mandible during mouth opening [15]. 

Intervention 

Both studies included a systemic enzyme therapy combination of trypsin, rutoside, and bromelain.

Comparison 

In the study conducted by Jayachandran and Khobre, 30 patients with TMJ osteoarthritis were divided into three groups: diclofenac sodium alone (50mg twice a day), oral enzymes (bromelain, trypsin, and rutoside trihydrate), and a combination of both [14]. In the study conducted by Gupta et al., 30 patients with internal derangement (ID) were included in two groups. These patients received diclofenac sodium 50mg (Diana D, Mapra) twice daily for 14 days or a combination of trypsin 48mg, bromelain 90mg, rutoside trihydrate BP 100mg, and diclofenac sodium 50mg (Zymoflam D, Aristo Pharma) twice daily for 14 days. Additionally, they were advised to perform jaw exercises and apply moist heat. This group served as the comparison group in the study [15].

Outcomes 

In the studies conducted by Jayachandran and Khobre, a p-value of 0.05 indicated that patients receiving systemic enzymes along with NSAIDs exhibited better responses compared to Group 1 and Group 3. Conversely, when comparing systemic enzyme treatment with systemic enzymes combined with NSAIDs, a p-value greater than 0.05 suggested that both groups responded equally to the treatment. In the trial, individuals receiving oral enzymes and diclofenac sodium combination therapy showed a significant improvement in pain reduction [14].

According to Gupta et al.'s findings, both groups experienced less pain, better eating and mouth opening, as well as a decrease in joint noise and jerky mandibular motions. Patients receiving systemic enzyme therapy performed better than those receiving systemic enzyme therapy, and the differences were quite substantial. For the treatment of internal derangement of the TMJ, we advise combining enzymes and diclofenac [15].

Discussion 

Patients with temporomandibular joint disorder (TMD) are more likely to have pain, TMJ noises, and incorrect mandibular motion [14]. The jaw, masticatory muscles, and TMJ noises are frequently the only places where the pain is felt. Tinnitus, ear pain and stuffiness, neck pain, dizziness, and headaches are typical interconnected symptoms associated with certain conditions affecting the ear, such as TMJ disorders or other ear-related issues. In some cases, the condition may present acutely, with symptoms being minor and temporary. In certain individuals, chronic TMD can develop, characterized by persistent pain and a range of physical, psychological, behavioral, and psychosocial symptoms. These symptoms are akin to those observed in patients with chronic pain syndromes affecting other body parts, including chronic headache, chronic regional pain syndrome, arthritis, low back pain, and fibromyalgia. Consequently, a comprehensive diagnostic approach and treatment strategy are essential for managing such cases [15-17]. 

Svarney in Physiopedia mentioned that the human body has roughly 75,000 different kinds of enzymes. They are crucial for inflammatory processes as well as other processes. in connection with the immunological system as a defensive mechanism. In doing so, the body endeavors to safeguard its long-term health [16, 17]. Proteolytic enzymes such as pancreatin, papain, rutin, trypsin, and chymotrypsin are crucial regulators and inflammatory response modulators.

Jayachandran and Khobre [14] showed a higher prevalence of TMJ osteoarthritis in females, similar to studies reported by Alexiou et al. [18]. The occurrence of osteoarthritis increases with age.

As a result of its propensity to inhibit neutrophil migration and the release of pro-inflammatory cytokines, bromelain has anti-inflammatory characteristics. Trypsin is known to exhibit antioxidative effects, and it can influence the activation of protease-activated receptor 2. This action has been associated with a reduction in the inflammatory reaction [17]. Rutoside trihydrate has been reported to have anti-inflammatory properties, and it is suggested that it can inhibit the production of pro-inflammatory genes in human macrophages [10]. The oral enzymes prescribed were 100mg of rutoside trihydrate, 90mg of bromelain, and 48mg of trypsin. It was employed in the form of an enteric-coated dosage and is considered an acceptable tablet. Patients in this study, who were between the ages of 40 and 60, were included with an average age of 49 years. The typical age was found to be similar to that observed in other studies. Studies have shown that proteolytic enzymes can be comparable to, or even more effective than, potent steroids and non-steroidal anti-inflammatory medications like phenylbutazone, hydrocortisone, indomethacin, and acetylsalicylic acid. Moreover, the benefits of proteolytic enzymes seem to be enhanced when these enzymes are combined [16].

Upon further follow-up, the study revealed a highly significant decrease in pain for both groups. Moreover, a substantial difference in pain reduction between the first and second follow-ups of patients in Groups 1 and 2 was observed, indicating that patients in Group 2 experienced faster pain alleviation compared to those in Group 1. The patient's ability to chew improved in both groups on subsequent follow-up visits. Patients in Group 2 demonstrated a faster improvement in chewing skills compared to Group 1, and this difference was statistically significant. Furthermore, in subsequent trials, both groups exhibited an increase in mouth opening.

The absence of a discernible difference in follow-up among the research groups suggests that painkillers may have a substantial impact on the factors contributing to oral pain and may provide relief to the masticatory muscles. Oral enzyme preparations exhibited comparable efficacy and tolerability and were demonstrated to be similarly effective as diclofenac, based on the treatment response observed in all groups. This suggests that oral enzyme preparations can be considered as an alternative with similar therapeutic outcomes as diclofenac in the context of the study.

Studies have reported that Consolidated Standards of Reporting Trials (CONSORT) guidelines should be followed while reporting randomized controlled trials. The clinicians should minimize harm and provide maximum benefits to the patients. Longer post-treatment follow-up is required to analyze the performance of systemic enzyme therapy on chronic TMJ diseases [14, 15, 19].

Limitations 

Very few randomized controlled trials are done on systemic enzyme therapy and its effect on TMJ disorders. Although similar studies exist with treatment for knee arthritis and wound healing, a moderate amount of bias concerning blinding and randomization exists. As the pain score is subjective, it is difficult to generalize the results.

## Conclusions

Limited literature is available on the clinical efficacy of systemic enzyme therapy for diseases of the temporomandibular joint. However, the analyzed studies showed improvement and were statistically significant concerning the study population. The efficiency of trypsin, bromelain, and rutoside is superior to conventional NSAIDs. More studies need to be conducted and reviewed on this topic to enhance our understanding of the complex pathogenesis and management of TMJ diseases.
